# Reconstruction of Bilateral Chronic Triceps Brachii Tendon Disruption Using a Suture-Mediated Anatomic Footprint Repair in a Dog

**DOI:** 10.3390/ani14111687

**Published:** 2024-06-05

**Authors:** Jong-Pil Yoon, Hae-Beom Lee, Young-Jin Jeon, Dae-Hyun Kim, Seong-Mok Jeong, Jae-Min Jeong

**Affiliations:** Department of Veterinary Surgery, College of Veterinary Medicine, Chungnam National University, 99, Daehak-ro, Yuseong-gu, Daejeon 34134, Republic of Korea; yjp0101@naver.com (J.-P.Y.); seatiger76@cnu.ac.kr (H.-B.L.); orangee0115@gmail.com (Y.-J.J.); vet1982@cnu.ac.kr (D.-H.K.); jsmok@cnu.ac.kr (S.-M.J.)

**Keywords:** chronic triceps brachii tendon disruption, footprint repair, suture-mediated anatomic repair, Krackow suture, trans-articular external skeletal fixation, dog

## Abstract

**Simple Summary:**

Chronic triceps brachii tendon disruptions in dogs can lead to significant lameness and discomfort, often requiring surgical intervention for effective treatment. This case report details the surgical reconstruction of bilateral chronic triceps brachii tendon disruptions in a 2-year-old female Pomeranian using a novel suture-mediated anatomic footprint repair technique. The technique, adapted from human medicine, involves creating a precise attachment of the tendon to the olecranon through specialized suturing and bone tunneling, which aims to restore normal anatomy and function. Following the surgery, the dog experienced significant improvement in forelimb function and was able to maintain a normal gait over a three-year follow-up period. This report demonstrates the successful application of a human surgical technique in veterinary medicine, providing a promising option for managing this rare but challenging condition in dogs. The technique’s success suggests its potential utility in similar cases, offering insights that could benefit surgical practices in veterinary orthopedics.

**Abstract:**

A 2-year-old, intact female Pomeranian presented with bilateral forelimb lameness, characterized by the olecranon making contact with the ground. The patient experienced two separate incidents of falling, occurring four and three weeks before admission, respectively. Following each episode, non-weight-bearing lameness was initially observed in the left forelimb, followed by the development of crouch gait. Based on the physical examination, radiographic, and ultrasonographic findings, bilateral triceps brachii tendon disruption was diagnosed. Intraoperatively, excessive granulation tissue at the distal end of the tendon was excised. The footprint region of each triceps brachii tendon was decorticated with a high-speed burr until bleeding was observed. The triceps brachii tendon was reattached to completely cover its footprint on the olecranon using the Krackow suture technique. This method involves anchoring the suture through bone tunnels in the ulna. Trans-articular external skeletal fixation was applied to both forelimbs to immobile and stabilize the elbow joints for nine weeks. Subsequently, the dog gradually increased its walking activities while on a leash over a six-week period. At the three-year follow-up, the patient exhibited improved forelimb function and maintained a normal gait without signs of lameness. Suture-mediated anatomic footprint repair proved useful in this single case and may be an effective surgical alternative for the management of chronic triceps brachii tendon disruption in dogs.

## 1. Introduction

Disruption of the triceps brachii tendon, characterized by the tearing or detachment from its insertion on the olecranon, is rarely reported in small animals [1,2,3,4,5]. The etiology of this condition in small animals typically involves trauma, surgical interventions near the tendon attachment, and local or systemic corticosteroid injections, with trauma being the predominant cause [1,2]. Clinically, this condition is often manifested by non-weight-bearing lameness, soft tissue swelling, and pain upon palpation [1,2,4].

Diagnosis of triceps brachii tendon disruption relies on comprehensive physical and imaging examinations [1,2,6]. Physical examination may reveal a nodular formation and a palpable gap proximal to the olecranon of the affected limb. It is noteworthy that the squeeze test, which assesses tendon injury, may often be inconclusive [7]. Radiographic evaluations typically identify a flake sign at the triceps brachii tendon attachment, indicative of a potential avulsion injury [1,4]. Ultrasonography generally shows discontinuity or an abnormal appearance of the tendon fibers [5]. Magnetic resonance imaging (MRI) is considered the preferred method for diagnosing and differentiating complete from partial triceps brachii tendon disruption [7,8].

Given that tendon disruptions rarely heal with conservative therapy, surgical intervention is the standard treatment [2,9]. These disruptions can be categorized into three patterns as follows: myotendinous junction, central part of the tendon, and osseo-tendinous junction [1]. Disruption at the central part requires a tendon–tendon suture, while osseo-tendinous disruption necessitates a tendon–bone suture through bone tunnels [1,10]. Techniques for tendon–bone sutures include trans-osseous, suture anchor, and hybrid trans-osseous–suture anchor approaches [9,11]. The restoration of the anatomical footprint and the prevention of postoperative gap formation are crucial for tendon healing, as they significantly impact the biomechanical strength and speed of recovery [11,12,13]. In human medicine, traditional cruciate repair techniques utilized for tendon restoration have demonstrated a 21% recurrence rate because of the limited contact area between the tendon and bone. This limited contact reduces mechanical strength and delays healing. Consequently, techniques employing suture anchors are preferred for their strong holding strength and ease in reconstructing the footprint, which leads to successful outcomes [9,11].

Such footprint restoration techniques, while established in human medicine, have not been commonly applied in veterinary medicine, especially for small-breed dogs.

The application of anchors in small dogs can be challenging because of the limited bone availability at the olecranon [9]. Moreover, in chronic cases, excessive scar tissue, tendon retraction, and muscle atrophy can occur following tendon degeneration, resulting in a large gap [3,14,15,16,17]. The poor tissue quality of tendon ends may also adversely affect the suture’s holding strength [3]. Therefore, a trans-osseous technique that can mechanically withstand long-term stress and prevent gap formation while reconstructing the footprint seems indicated for dogs with chronic triceps brachii tendon disruption. This report describes the successful outcome of surgical treatment using a novel suture-mediated anatomic footprint repair in a dog with chronic bilateral triceps brachii tendon disruptions.

## 2. Case Description

**Figure 1:**
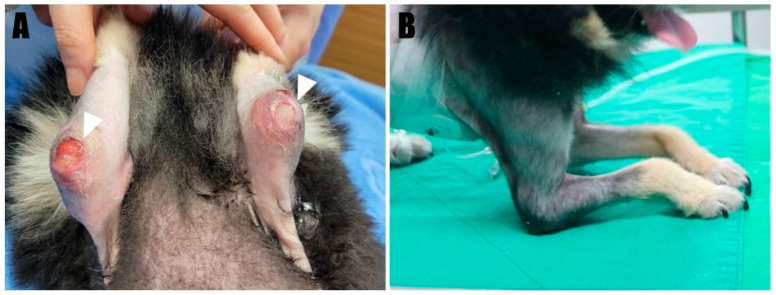
(**A**) Chronic ulceration wounds were evident on the bilateral elbow (arrowhead). (**B**) The dog exhibited a crouching posture, indicative of an abnormal standing.

**Figure 2:**
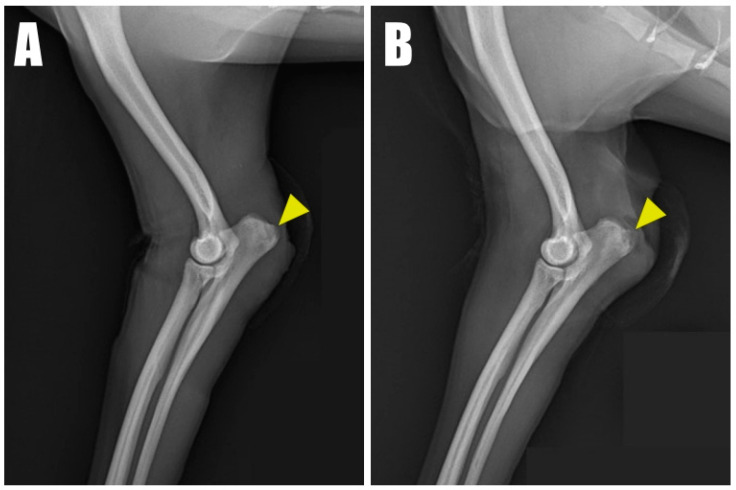
Radiolucent opacity (yellow arrowhead) was visible on the olecranon in radiographic images of the (**A**) right and (**B**) left forelimbs.

**Figure 3:**
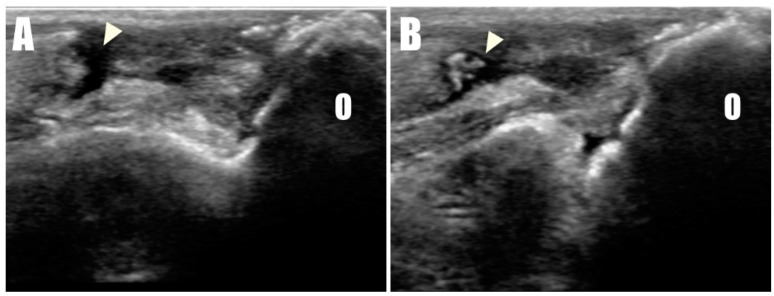
Disruption of the triceps brachii tendon (arrowhead) from the olecranon (O) was confirmed in ultrasonographic images of the (**A**) right and (**B**) left sides.

A 2-year-old, 4.5 kg, intact female Pomeranian was referred to a veterinary medicine teaching hospital with bilateral open wounds (Video S1, Figure 1A) at the olecranon region. The dog presented with a history of bilateral forelimb lameness and crouch gait (Figure 1B) as follows: four weeks in the left forelimb and three weeks in the right forelimb, each following a fall from a height. Physical examination revealed a pain response and nodular formation in the proximal region of the olecranon, with no extension response during the triceps brachii squeeze test. Radiographs revealed radiolucent opacities on both sides of the olecranon (Figure 2A,B), and ultrasonography identified defects (Figure 3A,B) at the triceps brachii tendon and olecranon junction, accompanied by inflammation and edema. Notably, the distal end of the proximal tendon appeared hyperechoic compared with the normal tendon. Based on the diagnosis of bilateral triceps brachii tendon disruption, believed to be of traumatic origin, and with the exact cause remaining open to interpretation, surgical repair was indicated. The initial management of the open wounds involved sugar dressing and debridement. Pre-anesthetic evaluation through blood samples, assessing electrolytes, and complete blood count (CBC), revealed all values within normal limits.

The surgical procedures were performed with informed consent from the owner. The dog was premedicated with midazolam (0.2 mg/kg, IV) and ketamine (0.5 mg/kg, IV), followed by induction with propofol (6 mg/kg, IV) and maintenance of general anesthesia with isoflurane and oxygen via a rebreathing circuit. Analgesia was provided by constant rate infusion of remifentanil (0.1–0.3 ug/kg), and cefazolin (22 mg/kg, IV) was administered for prophylaxis 30 min before incision and repeated every 90 min for a total of three doses.

**Figure 4:**
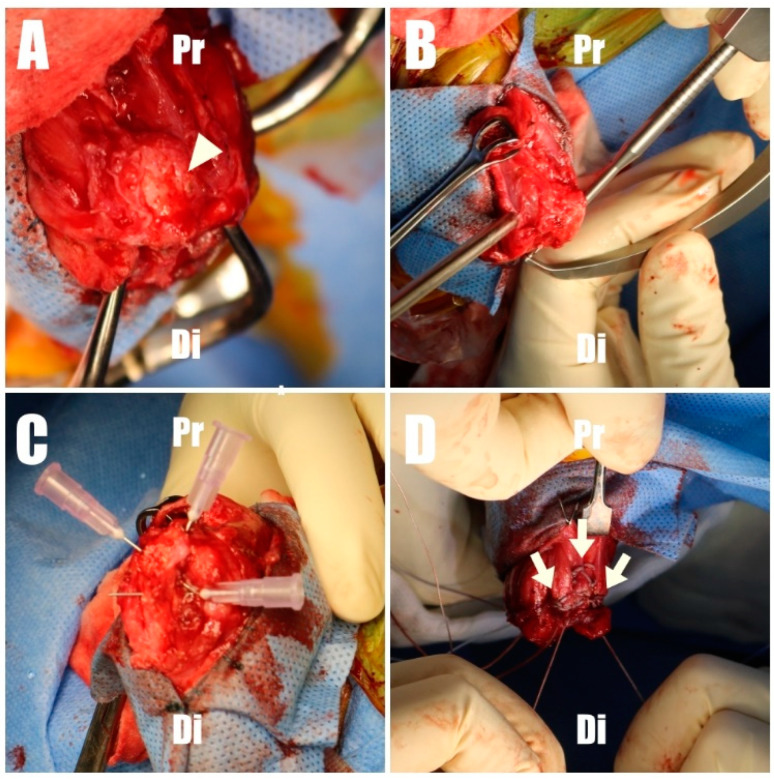
(**A**) The footprint (arrowhead) of the olecranon was decorticated to promote tendon-to-bone healing. (**B**) A C-guide was employed to create a caudal suture hole at the footprint. (**C**) The subsequent process involved the careful marking of the suture hole positions for verification using 26G needles. (**D**) The final stage involved the application of three Krackow stitches (arrow) for tendon repair with 2-0 FiberWire. Pr, proximal; Di, distal.

**Figure 5:**
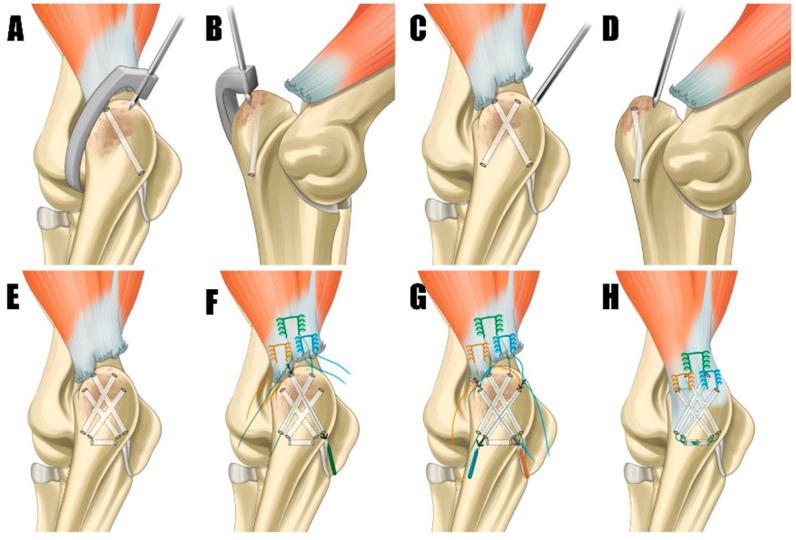
(**A**,**B**) These tunnels were originated cranially, employing a C-guide to maintain the integrity of the cross pattern. (**C**,**D**) Kirschner wire was used to meticulously create bone tunnels in a cross pattern within the footprint region, directed in craniomedial and craniolateral orientations for suture placement. (**E**) The final bone tunnel was created transversely along the ulna’s long axis. (**F**) The application of three Krakow sutures to the tendon, with the most proximal suture (green) passed through the cranial bone tunnel using a suture passer, exiting laterally and medially to enable crossing. (**G**) Two distal sutures (brown and blue) were passed through the caudal bone tunnels. Each suture’s inner strand was threaded through the bone tunnel on the opposite side to create a crossing configuration. (**H**) All sutures were passed and crossed through the transverse bone tunnel, forming a knot.

The dog was placed in dorsal recumbency with bilateral elbow joints placed uppermost. To approach the humeroulnar part, a caudolateral approach to the olecranon was made [2]. Fascia were incised and retracted, exposing the triceps brachii tendon and olecranon. Granulation tissue was meticulously debrided with a rongeur and scalpel blade, and the triceps brachii footprint (Figure 3A) on the olecranon was decorticated using an electrically powered oval-shaped burr (Core, Stryker, Kalamazoo, MI, USA) to promote tendon-to-bone healing [12]. The bone tunnel preparation and suture repair technique were adopted from previously reported all-suture repair methods in human medicine, with slight modification. Initially, the origins of the anconeus and flexor carpi ulnaris were elevated from the olecranon to facilitate proper bone tunneling (Figure 4A). Subsequently, two tunnels were created on the craniomedial and craniolateral aspects of the footprint. These tunnels were drilled at 40˚ relative to the proximal anatomic axis of the olecranon, directed caudodistally, using an aiming device and a 1.2 mm Kirschner wire (K-wire) (Figure 4B and Figure 5A,B). The tunnels were drilled to intersect in a “cross” pattern. Similarly, two additional tunnels were created cranially compared to the initial pair, angled approximately 60 degrees to the proximal anatomical axis of the olecranon, to maintain the cross pattern (Figure 4B and Figure 5C,D). The final bone tunnel was drilled transversely across the long axis of the ulna using a 1.6 mm K-wire, serving as an anchorage point for the suture knots (Figure 4C and Figure 5E).

A 2-0 size FiberWire suture (Arthrex, Naples, FL, USA) was employed using a locking Krackow technique (Figure 4D), positioned at the proximal aspect of the tendon. This ensured that enough tendon distal to the suture exits was left to completely cover the triceps brachii footprint. The suture ends were passed through the most craniomedial and craniolateral bone tunnels in a crossing pattern, with the lateral suture end entering the craniolateral hole and exiting caudomedially, and the medial suture ends entering the craniomedial hole and exiting caudolaterally (Figure 5E). Second and third locking Krackow sutures were placed parallel on each medial and lateral side of the triceps brachii tendon’s distal end (Figure 5F). The medial ends of each suture were passed through the caudally positioned craniomedial hole, and the lateral ends through the caudally positioned craniolateral hole, using a suture passer. Three limbs of the suture were threaded through the transverse tunnel (Figure 5H). The suture exiting the proximal aspect of the tendon was tied first, with the elbow in full extension to ensure anatomical reconstruction. After the initial tie, the sutures emerging distally were matched and tied accordingly.

Saline lavage was performed on the surgical site, which was then swabbed for culture and sensitivity tests before routine closure. Following closure, a trans-articular external skeletal fixator (TAESF) was applied for joint immobilization. A circular ring was placed on the radius using two 1.0 mm K-wires. Two 2.4 mm Duraface ESF end thread pins (IMEX Veterinary InC., Longview, TX, USA) were inserted into the humerus and connected with lateral connecting bars. The elbow joint angle was immobilized at 150 degrees to reduce tension on the tenorrhaphy. The contralateral limb underwent identical treatment.

**Figure 6:**
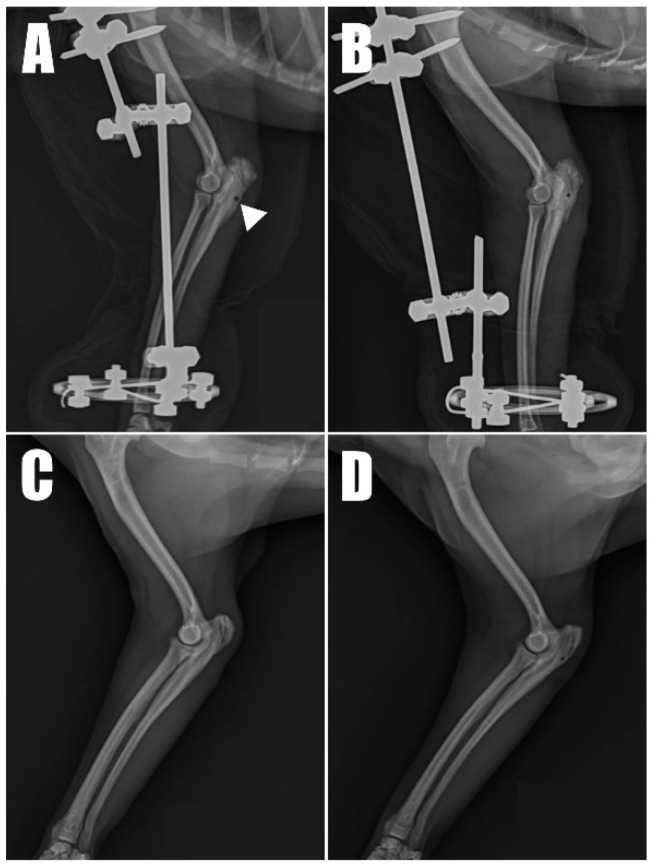
Immediate postoperative radiograph of the (**A**) right forelimb showing the transverse hole (arrowhead) in the caudal aspect of the ulna. (**B**) A corresponding image of the of the left forelimb. Six-month postoperative radiograph of the (**C**) right and (**D**) left forelimb.

**Figure 7:**
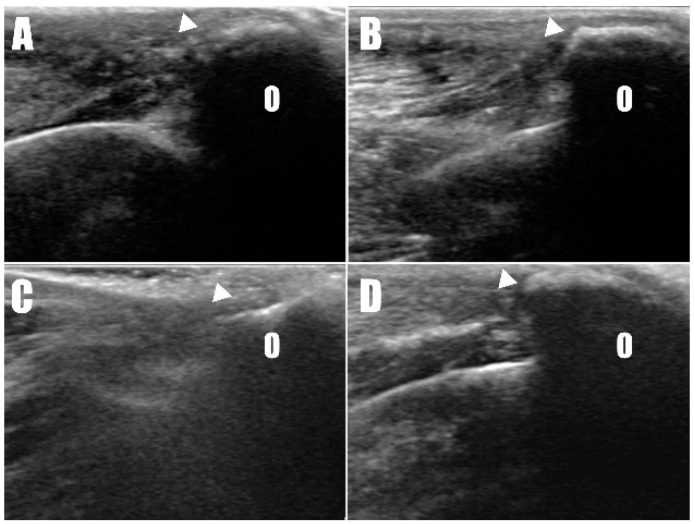
Ultrasound images taken two weeks postoperatively of the (**A**) right and (**B**) left forelimbs confirm the attachment of the triceps brachii tendon (arrowhead) to the olecranon (O). Similar findings were observed in the ultrasound images six months postoperatively on the (**C**) right and (**D**) left forelimbs.

Postoperative radiographs and ultrasound images (Figure 6) were taken to confirm the position and connectivity (Figure 7) of the tendon on the olecranon. On the right side (Figure 6A), the transverse hole was observed to be close to the caudal cortex, while the remaining bone tunnels were confirmed to have been drilled as intended. For analgesia, remifentanil was continued for three days postoperatively at 0.1~0.3 ug/kg/min with constant rate infusion. The other postoperative therapy including antibiotics (amoxicillin and clavulanic acid, 12.5 mg/kg BID, for 14 days/Clindamycin, 11 mg/kg BID, for 14 days), NSAID (meloxicam, 0.1 mg/kg SID for 14 days), and gastrointestinal protectant (esomeprazole, 1 mg/kg SID for 14 days) were administered.

Immediate postoperative weight-bearing was observed. The patient was discharged two weeks postoperatively with instructions for cage rest. To prevent joint ankylosis, the bolts and nuts were slightly loosened, and the trans-articular external skeletal fixator (TAESF) was progressively released six weeks postoperatively to allow a limited range of motion of about 10 degrees. The range of motion (ROM) was gradually increased to a maximum of 30 degrees, and ultimately, the TAESF was removed nine weeks postoperatively. The limbs were then supported in a Spica splint for an additional two weeks.

**Figure 8:**
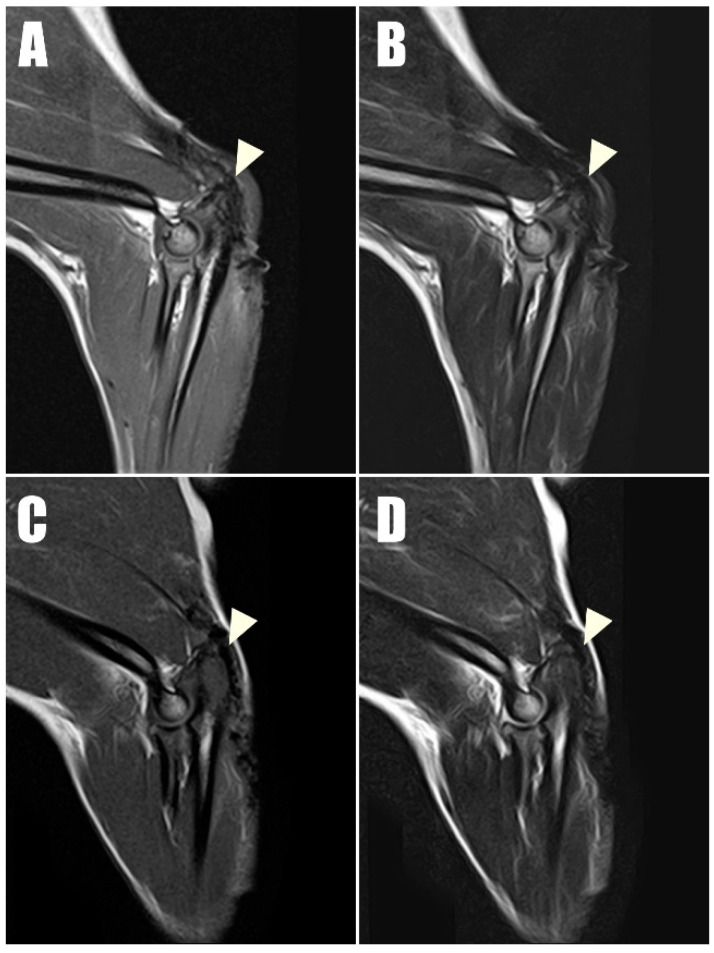
Postoperative magnetic resonance imaging (MRI) taken one year postoperatively captured (**A**) T1-weighted and (**B**) T2-weighted sagittal images of the right triceps brachii tendon, along with (**C**) T1- T1-weighted and (**D**) T2-weighted sagittal images of the left of triceps brachii tendon. These images demonstrate low signal intensity on both T1 and T2 sequences, indicating intact connectivity of the tendon.

Radiography (Figure 6C,D) and ultrasonography (Figure 7C,D) six months postoperatively confirmed that the transverse hole of the right side healed without any complications, and the bilateral triceps brachii tendons were well-maintained and attached to the olecranon, with no specific changes in internal echogenicity or echotexture. MRI (Figure 8) showed mild inflammatory changes near the suture knot and fibrotic scar tissue within the tendon but confirmed firm attachment of the tendon to the olecranon. Three years following the surgery, the patient exhibited no signs of functional loss (Video S2) or pain, confirming the long-term success of the treatment. The owner expressed satisfaction with the clinical outcomes.

## 3. Discussion

A suture-mediated footprint anatomic repair of the triceps brachii tendon was successfully utilized to restore full and recurrence-free forelimb function in a dog. The significance of this case lies in its novel application of a suture technique, previously documented in the human medical literature but adapted here for veterinary use with modification. The procedure resulted in excellent limb function as evidenced by long-term follow-up. This positive outcome, although in only a single case, is encouraging, and the technique can be recommended in similar cases.

Triceps brachii tendon disruption, though rare in small animals, poses significant challenges because of its varied etiology and the complexity of surgical repairs [1,2,3,4,5]. In humans, triceps brachii tendon injuries are attributed to multiple factors, including trauma, anabolic steroid use, weightlifting [18], local steroid injections [19], and a range of systemic conditions. Similarly, in veterinary medicine, tendon disruptions have been reported following local steroid injections [5], and breed-specific differences cannot be ruled out. Notably, triceps brachii tendon disruptions have been observed in Pomeranians [2,4], highlighting the need for further research to understand potential breed-specific predispositions. In our Pomeranian case, the bilateral nature of the tendon disruption, precipitated by falls, does not entirely exclude the possibility that degenerative changes, similar to those seen in cranial cruciate ligament disease, were exacerbated by minor trauma [20]. Additionally, inadequate vascularization [21] or a smaller insertion site at the tendon’s insertion compared with other regions could also be contributing factors to this disruption. Further research is needed to explore these aspects comprehensively.

In human medicine, triceps brachii tendon disruptions typically occur at the osseo-tendinous junction, where several repair strategies are employed, including trans-osseous, suture anchor, and hybrid approaches [10,22]. However, traditional trans-osseous repairs, which have a notable failure rate of approximately 21%, often fail to adequately cover the triceps brachii footprint, leading to higher failure rates compared with those using suture anchors [9,11]. These anchors, however, present limitations in small dogs because of the risk of bone fracture [9]. Importantly, anatomic footprint repairs have demonstrated the highest average failure yield load, emphasizing their effectiveness. Specifically, when comparing gap formation rates, which are crucial for the tendon’s healing process, traditional methods exhibit a high rate of 69%, suture anchors have a rate of 52%, and anatomic footprint repairs show a significantly lower rate of 14% [13,17]. In light of these factors, the consideration of anatomic footprint repair and meticulous surgical preparation is essential when addressing tendon disruptions at the osseo-tendinous junction [9]. In this case, a suture-mediated anatomic footprint repair technique, developed for human medicine, was adapted for a chronic condition in a dog, utilizing triple Krackow stitches and modifying the attachment method with two additional holes. This modification expanded the attachment surface area, enhancing durability and reducing gap formation.

The decision to use a trans-articular external skeletal fixator (TAESF) over external coaptation was influenced by the dog’s history of pressure sores and the need for surgical wound access. TAESF, typically applied for three to eight weeks, in this case, was maintained for approximately nine weeks because of the chronic bilateral rupture, which anticipated a slow healing process. Adjustments were made six weeks postoperatively to prevent elbow joint ankylosis by allowing limited movement.

Tendon structural integrity and musculotendinous change are best evaluated by magnetic resonance imaging (MRI). In the late stages of tendon healing, tendons typically exhibit low signal intensity across T1- and T2-weighted MRI sequences [23]. In the current case, the bilateral triceps brachii tendons at their attachment to the olecranon demonstrated low signal intensity in all sequences at the one-year postoperative evaluation, with the exception of mild inflammatory changes observed near the suture knots and surrounding scar tissues.

This case report, while offering some insights, is constrained by several limitations. Firstly, it is based on a singular case, which may limit the broader applicability of our findings. Without the support of more extensive diagnostic procedures, such as a biopsy, we acknowledge our limitations in conclusively identifying other potential causes. Furthermore, there is a recognized need for more comprehensive biomechanical studies to compare the efficacy of traditional repair techniques, suture anchors, and the anatomic footprint repair technique applied in this case. Finally, the investigation into the triceps brachii tendon’s footprint area in dogs remains an uncharted area of study, suggesting a valuable opportunity for future research to fill this gap in the veterinary literature.

## 4. Conclusions

In conclusion, this case report details the surgical management and postoperative care of a dog with chronic bilateral triceps brachii tendon disruption, illustrating the importance of suture-mediated anatomic footprint repair combined with trans-articular external skeletal fixation (TAESF). It underscores the critical role of understanding and preserving the tendon’s footprint during reconstruction for successful recovery. However, it also acknowledges the gap in our current knowledge regarding the precise extent of the triceps brachii tendon’s footprint area in dogs, emphasizing the need for further research in this area to enhance surgical outcomes in similar cases.

## Data Availability

The original contributions presented in this study are included in this article/the Supplementary Materials. Further inquiries can be directed to the corresponding author.

## Supplementary Materials

The following supporting information can be downloaded at https://www.mdpi.com/article/10.3390/ani14111687/s1, Video S1: Preoperative crouch gait. The olecranon made contact with the ground while walking. To prevent further damage to an open wound, the lib was supported with a sling during the gait assessment. Video S2: Postoperative gait. A normal gait has been consistently maintained for three years postoperatively.
